# Glucocorticoid Signaling in PSC-Derived Neural Systems to Elucidate Mechanisms of Stress-Induced Psychiatric Vulnerability

**DOI:** 10.1007/s12035-026-06041-1

**Published:** 2026-07-07

**Authors:** Eloiza Adriane Dal Molin, Manuella Pinto Kaster, Juliana Minardi Nascimento

**Affiliations:** Department of Biochemistry, School of Biological Sciences, Federal University of Santa Catarina, Florianopolis, SC Brazil

**Keywords:** Glucocorticoid signaling, Cortisol, Dexamethasone, HiPSC-derived neural models, Transcriptomic reanalysis, Stress-related psychiatric disorders

## Abstract

Glucocorticoids are major regulators of human neural development and stress adaptation, yet their transcriptional effects across experimental paradigms remain poorly integrated. This review synthesizes evidence from 13 studies employing human induced pluripotent stem cell (hiPSC)-derived neural models exposed to cortisol or dexamethasone, including neural progenitors, neurons, astrocytes, and brain organoids. When available, transcriptomic datasets from these studies were reanalyzed under standardized criteria to directly compare acute and chronic GC exposure. This comparative approach revealed that GCs modulate shared pathways related to neurogenesis, cytoskeletal organization, immune signaling, and stress response, while the duration of exposure critically shapes the underlying transcriptional architecture. Acute stimulation predominantly upregulated canonical GR targets such as FKBP5, ZBTB16, and TSC22D3 involved in early stress response and feedback control, whereas chronic exposure induced sustained remodeling of genes including MT2A, RASFAF4, and DPYSL5 linked to oxidative stress regulation and neuronal structure. A conserved downregulated core comprising NFIA, NFIB, and CCL2 was shared across paradigms, reflecting persistent suppression of glial differentiation and inflammatory signaling. Together, these findings delineate distinct yet convergent transcriptional programs governed by GC exposure, providing mechanistic insight into how temporal dynamics of glucocorticoid signaling may contribute to altered neurodevelopmental trajectories and increased vulnerability to stress-related psychiatric disorders.

**Supplementary Information:**

The online version contains supplementary material available at 10.1007/s12035-026-06041-1.

## Introduction to Glucocorticoids

Glucocorticoids (GCs) are essential steroid hormones that regulate energy metabolism, immune responses, and stress adaptation. Their secretion is dynamically controlled by the hypothalamic–pituitary–adrenal (HPA) axis, the primary central regulator of stress responses [1]. In the central nervous system, physical stressors often strongly engage brainstem and hypothalamic circuits involved in homeostatic regulation, whereas psychological stressors more prominently recruit cortical and limbic regions implicated in emotion, memory, and executive function, such as the amygdala, hippocampus, and prefrontal cortex [2–6]. Nevertheless, both physical and psychological stressors can activate overlapping neural circuits and converge on common downstream neuroendocrine responses, underscoring the integrative architecture of stress regulation in the brain.

In humans, cortisol is the predominant GC secreted by the adrenal cortex, with minor contributions from corticosterone [7, 8]. The physiological effects of GCs are mediated by two nuclear receptors: the glucocorticoid receptor (GR) and the mineralocorticoid receptor (MR), encoded by *NR3C1* and *NR3C2*, respectively. MRs, which have a higher affinity for cortisol (Kd ≈ 0.5–1 nM), are primarily expressed in the hippocampus and lateral septum, whereas GRs, which are activated at higher cortisol levels (Kd ≈ 5–10 nM), are widely distributed in the brain, including the hippocampus, hypothalamic paraventricular nucleus, and anterior pituitary [6, 9].

Beyond receptor abundance, both *NR3C1* and *NR3C2* undergo alternative splicing and post-translational modifications that generate multiple isoforms with distinct regulatory functions. Among these, GRα is the canonical ligand-activated form responsible for most transcriptional responses to GCs, while GRβ and other less abundant isoforms can exert dominant-negative or cell type–specific modulatory effects [10]. In neural cells, GRα expression predominates, although neuron–glia differences in GRβ and MR isoforms have been reported, suggesting that isoform-specific expression patterns may contribute to the heterogeneity of GC responses observed in vitro [11].

GR activity is further modulated by co-chaperones that determine receptor sensitivity and feedback strength. FKBP4 promotes GR activation by facilitating nuclear translocation, whereas FKBP5 impairs receptor sensitivity and feedback inhibition. Elevated FKBP5 expression has been repeatedly associated with GC resistance and stress-related HPA-axis dysregulation [12–15]. Under basal conditions, MR maintains tonic control of the HPA axis, while GR predominates during stress when cortisol levels rise.

In addition to these classical intracellular mechanisms, accumulating evidence demonstrates that GCs also signal through membrane-associated receptors, mediating rapid, non-genomic actions in neural cells. Jiang et al. [16] demonstrated that corticosterone activates both nuclear GC receptors, mediating genomic effects, and membrane-associated receptors that drive rapid modulation of synaptic transmission and neuronal excitability through transcription-independent mechanisms dependent on constitutive protein synthesis [16–19].

Integrating these experimental observations, Harrison and Tasker [20] proposed that GC signaling operates across multiple temporal scales through coordinated genomic and membrane-initiated pathways. In this framework, membrane-associated GR and MR (mGR and mMR) recruit diverse intracellular signaling cascades—including lipid and gaseous messengers, protein kinases, and receptor trafficking pathways—that dynamically regulate neurotransmitter release, synaptic efficacy, and cellular excitability, while also interfacing with nuclear GC signaling [20].

Consistent with this model, electrophysiological and live-cell imaging studies in hippocampal and amygdalar neurons have shown that rapid membrane-initiated GC actions facilitate presynaptic glutamate release and postsynaptic AMPA receptor trafficking, thereby enhancing excitatory synaptic efficacy and promoting long-term potentiation [20–22]. These rapid effects are frequently MR-dependent rather than GR-dependent and are sensitive to exposure dynamics, as prolonged or repeated corticosteroid exposure disrupts AMPA receptor trafficking and impairs long-term potentiation induction [20]. Collectively, these findings position membrane-associated GC signaling as a key regulator of synaptic plasticity and stress-dependent neural adaptation.

Synthetic glucocorticoids, such as dexamethasone (DEX), are widely used to experimentally probe GR-mediated mechanisms because they bind GR with 25–30-fold higher affinity than cortisol and exhibit minimal mineralocorticoid activity [9, 23–25]. DEX thus serves as a potent and selective tool for investigating GC signaling in vitro and in vivo. Pharmacokinetically, DEX is metabolized in hepatic microsomes via CYP3A4-dependent 6β-hydroxylation [26]. In healthy adults, it exhibits biphasic elimination, with a terminal half-life of 4–6 h. Despite this, suppression of endogenous cortisol persists for up to 24 h [27], reflecting extended receptor activation and genomic effects. This pharmacodynamic persistence is critical for defining exposure windows in vitro, where models aim to reproduce sustained GR activation rather than plasma clearance kinetics.

Despite its utility as a selective GR probe, the use of DEX to model clinical stress has important pharmacological caveats. Unlike endogenous cortisol, which maintains a balanced activation of both MR and GR, DEX exhibits negligible affinity for MR [28]. As a result, high doses of DEX can lead to a state of relative MR inactivity by potently suppressing endogenous cortisol secretion. This ‘MR depletion’ is clinically significant, as synthetic GC treatment in humans has been linked to an increased incidence of serious neuropsychiatric conditions, including psychosis, mania, and cognitive deficits [29]—side effects that are thought to arise from the loss of MR-mediated signaling and can be functionally rescued by MR agonists or low-dose cortisol add-ons [30, 31]. Consequently, findings obtained with synthetic GCs should be interpreted with caution, as they may not fully replicate the integrated MR/GR signaling profile of natural hormones.

Typical in vitro concentrations are based on clinically observed exposure ranges. Antenatal corticosteroid therapy, for example, yields maternal plasma concentrations of 162–245 nM and estimated fetal exposures of 65–98 nM [32, 33], supporting the use of ~ 100 nM DEX to model fetal brain exposure. Higher concentrations (100 nM–1 µM) are used to mimic pathological or stress-related states, corresponding to serum cortisol levels seen in Post-traumatic stress disorder (PTSD) (~ 493 nM; [34]).

Prolonged or excessive GC exposure—whether endogenous or pharmacological—induces broad systemic and neural effects. Chronic GC elevation, as observed in Cushing’s syndrome [35] and prolonged stress [36], leads to hippocampal atrophy, metabolic imbalance, and impaired HPA feedback [37]. Similarly, long-term corticosteroid therapy in humans has been linked to cognitive and affective disturbances [38], emphasizing that both intensity and duration of exposure shape GC-related outcomes. These systemic alterations parallel the cellular and molecular adaptations seen under chronic stress, including altered receptor signaling and gene regulation that contribute to long-term neural remodeling.

At the molecular level, chronic stress and prolonged GC exposure converge on pathways that regulate GR sensitivity and transcriptional control. Alterations in GR signaling and its molecular partners, particularly FKBP5 and NR3C1, have been consistently linked to impaired feedback regulation and abnormal cortisol dynamics in stress-related psychiatric disorders [39, 40]. PTSD is characterized by HPA hypoactivity and blunted cortisol secretion, whereas Major Depressive Disorder (MDD) typically presents with hypercortisolism and reduced GR responsiveness [41–43]. In anxiety disorders, cortisol patterns are more heterogeneous, reflecting variable feedback regulation [44].

Together, these findings illustrate that GC signaling dysregulation operates across multiple levels—from receptor dynamics to neural circuitry—underpinning vulnerability to stress-related psychiatric disorders. Understanding these mechanisms, including their cell-type–specific components, is essential for identifying novel therapeutic targets. Here, we integrate evidence from in vitro studies using human PSC-derived neural models and reanalyze transcriptomic datasets to identify GC-responsive molecular programs and their relevance to neuropsychiatric pathophysiology.

## Models for Studying Glucocorticoid Effects

There are multiple approaches to studying the effects of GCs on the brain, each with its own advantages and limitations. These models range from in vitro cell cultures to in vivo and ex vivo animal studies and postmortem human brain analyses. While each method provides valuable insights, none fully replicates the complexity of human neurobiology, making it essential to integrate different approaches to gain a more comprehensive understanding of GC-mediated processes [45].

Traditional in vitro models for studying GC effects include primary neural cultures derived from animal tissue, ex vivo brain slices, and immortalized cell lines of neuronal or glial origin. These systems enable tightly controlled experimental conditions and mechanistic investigations at the cellular and molecular levels but lack the structural, cellular, and functional complexity of the intact brain [23]. Importantly, such reductionist models are inherently unable to capture integrated circuit-level dynamics or behavioral phenotypes, which are central outcomes of GC action in vivo. Postmortem brain studies offer another means of examining GC-related alterations but are constrained by prolonged postmortem intervals, medication history, the limited availability of well-characterized samples, and the inability to perform longitudinal analyses, thereby complicating causal inference and temporal interpretation [46, 47].

Animal models have been widely used to investigate GC effects, particularly in neurodevelopment. Studies in rodents have demonstrated that prenatal exposure to synthetic GCs disrupts neurogenesis, alters neuronal differentiation, and contributes to behavioral alterations during adulthood [48]. However, species differences in neural progenitor cell composition, GR regulatory elements, and HPA-axis dynamics limit their translational relevance [49]. These discrepancies highlight the need for models that better capture human-specific GC effects.

To address these challenges, human-induced pluripotent stem cell (hiPSC)-derived models have emerged as a promising alternative. These systems provide a patient-specific platform to study GC-related pathophysiology while enabling genetic and epigenetic manipulations, high-throughput drug screening, and cost-effective experimental designs [23]. At the monoculture level, hiPSCs can be directed into key neural cell types used in GC studies, including neural progenitor cells [50], excitatory cortical-like neurons [51], GABAergic interneurons [52], astrocytes [53], oligodendrocytes [54], and microglia-like cells [55]. A notable advancement is the use of hiPSC-derived cerebral organoids (hCO), which replicate aspects of three-dimensional brain architecture and cell-to-cell interactions, closely resembling fetal brain development during early to mid-gestation—a critical period for neuronal migration, differentiation, and synaptic formation [48, 56, 57].

Despite their advantages, hiPSC and hCO models also have limitations. A major caveat is the absence of key brain-resident cell types in standard organoid systems, particularly microglia and functional vascular components, which play central roles in neurodevelopment, circuit maturation, metabolic support, and stress responsiveness [58, 59]. Microglia, in particular, dynamically interact with neural progenitors and synaptic networks and are increasingly recognized as modulators of developmental trajectories and neural circuit function, processes that are highly relevant for interpreting glucocorticoid effects [58, 60]. Although recent studies have incorporated iPSC-derived microglia or vascular-like structures into organoid platforms, partially mitigating these limitations, such systems still do not fully recapitulate the cellular complexity and functional integration of the human brain in vivo. Additional challenges include limited scalability, intrinsic cellular heterogeneity, and the restricted capacity of single-region organoids to model long-range interregional interactions, motivating the development of assembloid-based approaches [57, 61].

While no single model fully recapitulates the complexity of GC actions in the human brain, integrating in vivo and in vitro approaches remains essential. Advances in stem cell-based systems, combined with traditional models, continue to refine our understanding of GCs in neurobiology and disease [23].

## Glucocorticoid Modulation in Human Neural in vitro Models

To identify relevant studies evaluating GC effects in human neural in vitro systems, we performed a structured PubMed search between November 2024 and February 2025, using combinations of keywords such as “hiPSC”, (“hCOs” OR “cortical organoids”), “in vitro neural models”, AND (“glucocorticoid” OR “DEX” OR “cortisol”) AND (“psychiatry” OR “psychiatric” OR “stress”). This search retrieved studies across multiple human neural model types, including those derived from immortalized cell lines, primary fetal tissue, human embryonic stem cells (hESCs), and hiPSCs.

For the present analysis, we included only PSC–derived neural models—either hESC- or hiPSC-derived—while excluding immortalized or primary neural cell lines. This strategy enhances developmental relevance, as hPSCs can be directed to differentiate along neural lineage trajectories that recapitulate early human embryonic neurodevelopment including regional patterning and neural specification, in ways not feasible with conventional cell lines. Furthermore, hiPSC-derived models uniquely provide patient-specific genetic background, enabling the study of genetic influences on neurodevelopment and disease, which is particularly valuable in neuropsychiatric modeling [62].

Within this scope, we identified 13 original research articles investigating the effects of GCs—either cortisol or synthetic analogs—on PSC-derived neural cells. These models encompassed neural progenitor cells (NPCs), neurons, astrocytes, and hCOs, with heterogeneous treatment protocols and outcome measures.

Overall, the studies reveal a broad experimental spectrum, including both acute (hours) and chronic (days to weeks) GC exposures, with concentrations ranging from low nanomolar to micromolar levels. Acute treatments typically aimed to capture early GR-dependent transcriptional responses, while longer exposures highlighted sustained remodeling of gene expression and progressive changes in neural differentiation. Across models, DEX was most often employed as a selective GR agonist, whereas cortisol provided a more physiological comparison. Concentration- and time-dependent differences were consistently observed, with higher or prolonged exposures often reducing neuronal maturation and enhancing stress-related or gliogenic signaling profiles.

Together, these findings show that in vitro GC exposure differs substantially in duration and intensity, shaping distinct transcriptional and functional outcomes. Table 1 summarizes these studies, outlining the experimental parameters and key neural effects observed across different PSC-derived model types.

**Table 1:** Summary of glucocorticoid effects in human PSC-derived neural in vitro models

Study	Model	Treatment and concentration	Protocol	Main results
Neural progenitor cells				
Preynat-Seauve et al. [63]	hESC/ReNcell-VM-derived NPCs	DEX: 0.05 to 5 µg/mL (~ 0.127–12.74 µM)	6 days	DEX reduced neuronal differentiation and neurite outgrowth in NPCs
Ninomiya et al. [64]	hiPSC-derived NPCs	DEX, Bethamethasone or cortisol: 5 nM, 500 nM, and 50 µM	4 days	All GCs promoted NPC proliferation in a dose-dependent manner Cortisol increased neurons under H₂O₂-induced oxidative stress, while DEX and betamethasone increased total NPC numbers but not neuronal lineage cells
Nürnberg et al. [65]	hiPSC-derived NPCs	DEX: 5 nM, 50 nM, and 500 nM	14, 28 or 50 days	DEX increased NPC proliferation and inhibited neuronal differentiation in a dose-dependent manner at all time points measured
Neurons and Astrocytes				
Lieberman et al. [66]	hiPSC-derived forebrain-lineage mixed neural cultures (neurons and astrocytes)	DEX: 1 µM	6 h	*FKBP5* mRNA expression increased by 23%
Heard et al. [67]	hiPSC-derived astrocytes	Cortisol: 5 µM, and 50 µM	5 h	Cortisol increased expression of *ZBTB16* and *FKBP5*, and decreased IL1-β; similar effect at both doses
		Cortisol: 5 µM	24 h	GR expression was reduced; acute treatment upregulated *FKBP5*, *LYVE1*, and *DUSP1*
		Cortisol: 5 µM	7 days	Chronic treatment altered nearly twice as many genes as acute; regulated genes included *FKBP5, LYVE1, DUSP1*, among others; enriched pathways included cell adhesion, ECM organization, gliogenesis, and cell death
Seah et al. [34]	hiPSC-derived NGN2-induced glutamatergic neurons	Cortisol: 100 nM, 1 µM and 2.5 µM	24 h	Cortisol altered transcriptomic profiles without affecting cell density; Dose-dependent downregulation of genes related to acetylcholine signaling and upregulation of immune, cell signaling, and chromatin regulation pathways were observed. Neurite length decreased with increasing doses
Babaniyi et al. [68]	hESC-derived mixed neural cultures (neurons and astrocytes)	DEX: 0.1 nM, 1 nM, 10 nM, 100 nM, and 1 µM	6 h, 12 h	Only from DEX ≥ 10 nM (6 h) and 1 µM (12 h) increased *FKBP5; PER1* increased up to 100 nM but did not rise at higher doses
3D models				
Moors et al. [69]	hNPC-derived neurospheres	DEX: 1 μM	24 h	DEX reduced proliferation of neural progenitor cells and neuronal differentiation, increased astrocytic differentiation, and upregulated *DKK1*, leading to Wnt signaling inhibition
Notaras et al. [70]	hiPSC-derived forebrain organoids	Cortisol: 10 μg/mL (~ 27,6 μM)	7 days	Cortisol increased expression of proteins including NDUFB9, PBDC1, RBM28, FLYWCH2, ROCK2, NME7, RIF1, SLC16A2, A8BC6, ZNF579, PDLIM3, GGC2, and decreased expression of ARMC9, INTS9, VIRMA, NUP210; Cortisol decreased levels of 3-phosphoglyceric acid, creatinine, L-leucine, L-methionine, L-phenylalanine, L-tyrosine, N-acetylglutamine, N-acetylornithine, S-adenosylhomocysteine, and increased levels of guanosine triphosphate
Cruceanu et al. [48]	hiPSC-derived hCOs (30, 60, 90 DIV)	DEX: 10 nM, 100 nM, and 1000 nM	4 h, 12 h	DEX (10–1000 nM, 4 h and 12 h at 45 DIV) induced *FKBP5, TSC22D3, ZBTB16*; DEX (100 nM, 12 h at 30, 60, 90 DIV) upregulated *FKBP5, TSC22D3, MGARP*; downregulated *FOXG1, NEUROD6* Effects lasted ≥ 6 days after DEX removal
Byun et al. [71]	hESCs-derived hCOs (320 DIV)	Cortisol: 30 µM	7 days	Cortisol upregulated GR in astrocytes and increased MERTK expression in hCOs; It enhanced astrocytic engulfment of PSD95 + synaptic puncta and elevated LAMP2 + lysosome numbers, resulting in decreased global PSD95 expression
Krontira et al. [33]	hiPSC-derived hCOs (60 DIV)	DEX: 100 nM	10 days	DEX consistently led to a significant increase of PAX6⁺ EOMES⁺ basal progenitors and upper-layer SATB2⁺ neurons
Dony et al. [72]	hiPSC-derived hCOs (60 DIV)	DEX: 100 nM	10 days	Increased *GAD1*⁺ inhibitory neurons; DE in key TFs (*PBX3, YBX1, NFIB, SOX2*); *PBX3* linked to inhibitory neuron priming; increased *PBX3*⁺*GAD1*⁺ cells in transcript and protein
			10 days DEX + 20 days washout	12% overlap with the initial chronic treatment (10 days); persistent increase in neurons; sustained increase of *PBX3* expression
			10 days DEX + 20 days washout + 12 h DEX	61% overlap with chronic with washout treatment; 13% of genes respond differently depending on organoid’s history of chronic treatment

*hCOs* Human Cerebral Organoids, *hESC* Human Embryonic Stem Cells, *hiPSC* Human Induced Pluripotent Stem Cells, *A8BC6* ATP-binding cassette sub-family C member 6, *ARMC9* Armadillo Repeat Containing 9, *DEX* Dexamethasone, *DKK1* Dickkopf WNT Signaling Pathway Inhibitor 1, *DIV* Days in vitro, *DUSP1* Dual Specificity Phosphatase 1, *ECM* Extracellular Matrix, *EOMES* Eomesodermin, *FKBP5* FK506 Binding Protein 5, *FOXG1* Forkhead Box G1, *GAD1* Glutamate Decarboxylase 1, *GGC2* Germ Cell-Less, Spermatogenesis Associated 2, *GFAP* Glial Fibrillary Acidic Protein, *GR* Glucocorticoid Receptor, *IL-1β* Interleukin-1 beta, *INTS9* Integrator Complex Subunit 9, *LAMP2* Lysosome-Associated Membrane Glycoprotein 2, *LYVE1* Lymphatic Vessel Endothelial Hyaluronan Receptor 1, *MAP2* Microtubule-Associated Protein 2, *MERTK* MER Proto-Oncogene, Tyrosine Kinase, *MGARP* Mitochondria-localized Glutamic Acid Rich Protein, *NDUFB9* NADH:Ubiquinone Oxidoreductase Subunit B9, *NEUROD6* Neuronal Differentiation 6, *NFIB* Nuclear Factor I B, *NME7* NME/NM23 Family Member 7, *NPCs* Neural Progenitor Cells, *NGN2* Neurogenin 2, *NUP210* Nucleoporin 210, *PAX6* Paired Box 6, *PBX3* Pre-B-Cell Leukemia Homeobox 3, *PBDC1* Polysaccharide Biosynthesis Domain Containing 1, *PDLIM3* PDZ and LIM Domain 3, *PER1* Period Circadian Regulator 1, *PSD95* Postsynaptic Density Protein 95, *RBM28* RNA Binding Motif Protein 28, *RIF1* Replication Timing Regulatory Factor 1, *ROCK2* Rho Associated Coiled-Coil Containing Protein Kinase 2, *S100β* S100 Calcium Binding Protein Beta, *SATB2* Special AT-Rich Sequence-Binding Protein 2, *SOX2* SRY-Box Transcription Factor 2, *TSC22D3* TSC22 Domain Family Member 3, *VIRMA* Vir Like M6A Methyltransferase Associated, *YBX1* Y-Box Binding Protein 1, *ZBTB16* Zinc Finger and BTB Domain Containing 16, *ZNF579* Zinc Finger Protein 579

### Integrative Analysis of Global Effects of DEX

To integrate and expand upon the prior findings discussed above, we performed a comparative transcriptomic reanalysis of publicly available datasets involving DEX exposure in human neural models. This approach sought to reveal convergent transcriptional patterns that reflect the core molecular effects of GC signaling across different stages of neural differentiation and experimental conditions. Although both cortisol and DEX are commonly used in the literature, only DEX datasets provided supplementary material suitable for reanalysis. This integrative step therefore offers a complementary and global view of DEX-responsive genes and pathways, linking the heterogeneous observations described previously.

For this purpose, we gathered data from studies that made their processed expression matrices publicly available, including Cruceanu et al. [48], Babaniyi et al. [68], Krontira et al. [33], and Dony et al. [72]. Babaniyi et al. [68] analyzed a mixed population of neurons and astrocytes differentiated from hESC lines H1 and H9 after treatment with 1000 nM DEX for 6 h. Krontira et al. [33], Dony et al. [72], and Cruceanu et al. [48] investigated DEX effects in human cortical organoids (hCOs). Krontira et al. [33] and Dony et al. [72] evaluated chronic DEX exposure (100 nM for 10 days, starting at 60 days in vitro—DIV) in hCOs derived from hiPSCs lines HPS0076 and 409b2. Cruceanu et al. [48] assessed the response to 100 nM DEX for 12 h in hiPSC-derived neurons at 30, 60, and 90 DIV, derived from three independent hiPSC lines (including the reference lines HPS0076:409b2); results from these lines were analyzed together using the median expression values. All datasets were analyzed according to the data type reported by each study (bulk or single cell). Gene-level expression results were filtered to retain transcripts with FDR-adjusted *p*-values (or equivalent, such as *q*-values or padj) < 0.05 and absolute log2 fold change > 0.1. In cases where a gene was present in multiple datasets, the direction of regulation was determined by the record with the maximum absolute log2 fold change. Genes present in at least two independent studies were retained for subsequent analysis.

**Fig. 1:**
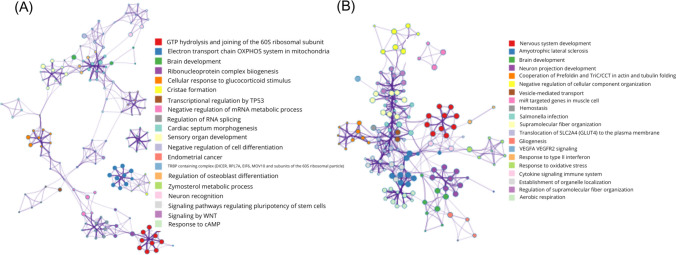
Functional enrichment networks of DEX-induced genes across human neural models. **A** Network of enriched biological processes among upregulated genes (*n* = 297) following DEX exposure in at least two datasets. **B** Network of enriched biological processes among downregulated genes (*n* = 392)

To gain insight into the biological pathways modulated by DEX, we submitted a list of 297 upregulated and 392 downregulated consensus genes (FDR < 0.05 in ≥ 2 studies) to Metascape [73] for functional enrichment analysis. The resulting ontology clusters, shown in Fig. 1, were visualized as interconnected networks and colored by cluster identity. In the upregulated gene network (Fig. 1A), the most prominent clusters involved GTP hydrolysis, ribosome biogenesis, and brain development, alongside a robust cellular response to glucocorticoid stimulus. This profile suggests an activation of protein synthesis machinery and developmental signaling in response to DEX. In contrast, the downregulated gene set (Fig. 1B) showed central enrichment for cell cycle, mitotic cell cycle, and negative regulation of cellular component organization, alongside cytokine signaling in the immune system. All enrichment results, including ontology terms, gene counts, and statistical metrics, are provided in Supplementary Table 1. Collectively, these results indicate a reproducible transcriptional shift across human neural models, characterized by the activation of steroid-responsive developmental programs and the coordinated suppression of proliferative and inflammatory pathways.

**Fig. 2:**
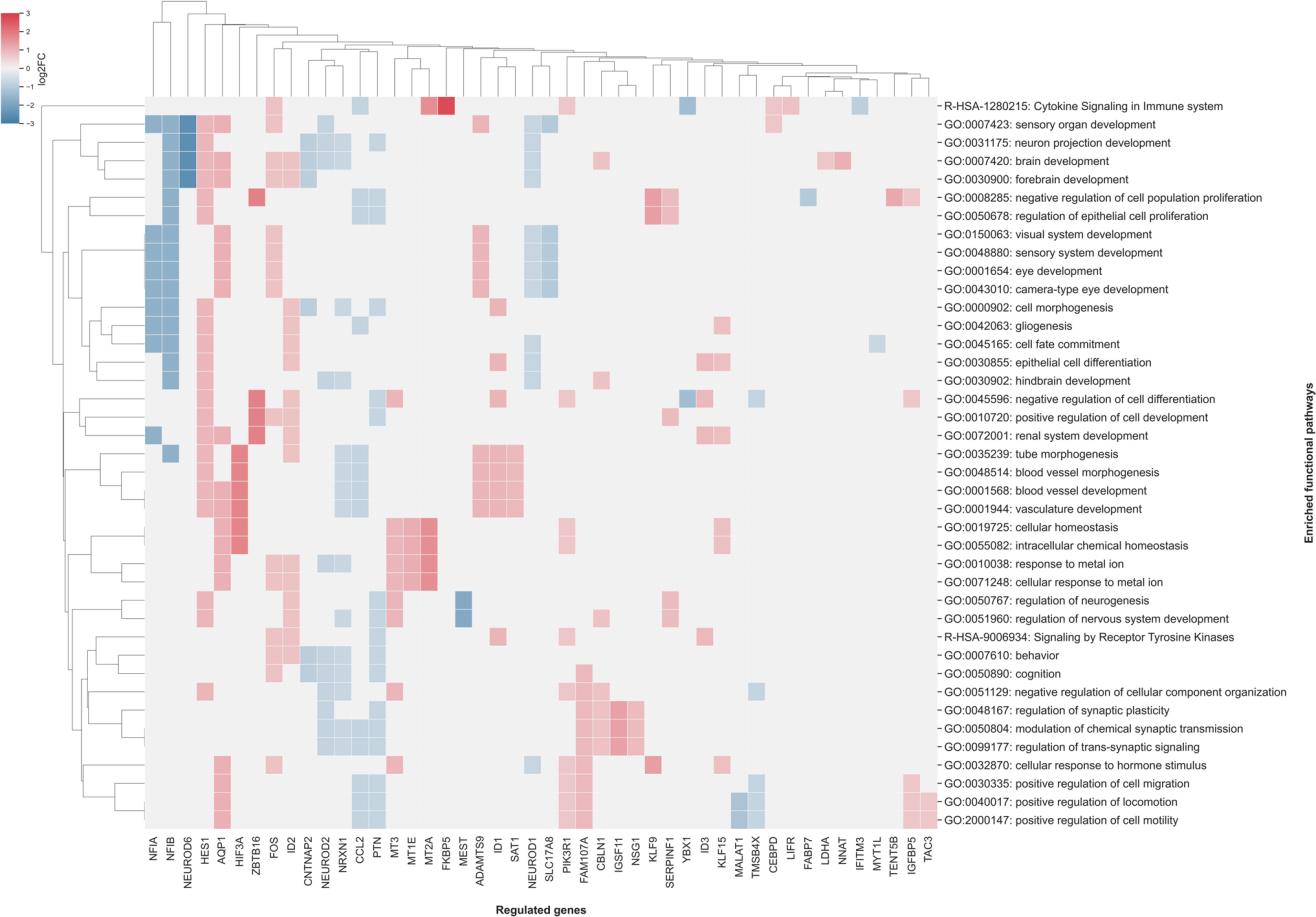
Clustered heatmap of top enriched biological processes and differentially expressed genes across DEX-exposed human neural models. Heatmap displaying the top 40 significantly enriched Gene Ontology biological processes (rows) and the 60 differentially expressed genes (DEGs) with the largest absolute expression changes across studies (columns). Functional enrichment was performed using Metascape, with gene–term associations filtered by a significance threshold of FDR/padj < 0.05. For genes present in multiple datasets, the record with the maximum absolute log₂ fold change (log₂FC) was selected. The displayed pathways were prioritized based on the density of associations (frequency) within the top 60 DEGs to highlight the most coordinated transcriptional responses. The color scale represents log₂FC values, with red indicating upregulation and blue indicating downregulation. Hierarchical clustering was applied to both genes and biological processes using Euclidean distance and complete linkage to identify co-regulated functional modules

To further explore these convergent transcriptional responses, we generated a clustered heatmap displaying the top 40 enriched biological processes and the top 60 DEGs showing the largest absolute changes in expression across studies (Fig. 2). To prioritize the most biologically representative results, functional pathways were selected based on their enrichment frequency (density of gene–term associations) specifically among these top 60 most regulated genes. This approach highlights processes where high-magnitude transcriptional changes are most concentrated, providing a refined view of coordinated responses. Within this heatmap, *HES1*, *AQP1*, and *FOS* emerge as highly upregulated genes, clustering with biological processes related to cellular homeostasis and response to hormone stimulus. In contrast, a prominent cluster of downregulated genes, including *NEUROD6*, *NFIA*, and *NFIB*, is associated with neuron projection development and broader neurodevelopmental programs, consistent with a coordinated repression of neuronal differentiation–related pathways following DEX exposure. A complete table listing all DEGs together with their associated functional terms and source studies is provided in Supplementary Table 2.

Consistent with the functional clustering observed in the heatmap, the downregulation of these core neurodevelopmental regulators—alongside others such as *GAP43* and *STMN2*—was enriched for terms related to neurogenesis and axon development, indicating a coordinated attenuation of neuronal maturation following DEX exposure. In contrast, consistently upregulated genes such as *PTN*, *ZBTB16*, *FOS*, and *ID2* mapped to biological processes involving gliogenesis and regulation of cell population proliferation, reflecting a shift toward adaptive and lineage-modulatory programs. Notably, several of these genes were linked to multiple functional terms, underscoring their pleiotropic regulatory roles across developmental and stress-related pathways. Together, these enrichment patterns suggest that DEX exposure in human neural in vitro models promotes transcriptional programs favoring stress adaptation and altered developmental trajectories, while concomitantly suppressing genes associated with neuronal differentiation and structural maturation.

To complement the heatmap-based analysis and to distinguish genes driven by large expression changes from those reflecting robust cross-study reproducibility, we employed a volcano plot integrating both the magnitude and the consistency of transcriptional regulation across datasets (Supplementary Fig. 1). This analysis highlights not only the most strongly up- and downregulated genes but also transcripts exhibiting concordant regulation in the same direction across all six datasets. Accordingly, genes such as *FKBP5*, *HIF3A*, *RASSF4*, *TSC22D3*, *PIK3R1*, *LIFR*, and *CHST15* are emphasized due to their reproducible upregulation across datasets, even when individual fold changes are moderate.

### Neural Progenitors

GCs modulate NPC fate through tightly regulated mechanisms involving receptor subtype, ligand specificity, intracellular context, and redox state. Across models, a consistent finding is that GC signaling promotes NPC proliferation while constraining their differentiation into mature neurons.

In human NPCs, Ninomiya et al. [64] demonstrated that DEX, betamethasone, and cortisol increased proliferation in a dose-dependent manner, both under basal conditions and oxidative stress. Notably, only cortisol—a ligand with higher affinity for the MR—significantly enhanced MAP2-positive neuronal differentiation, whereas DEX and betamethasone, which act preferentially via the GR, failed to do so. These findings suggest that while GR activation sustains NPC expansion, MR signaling may be required to promote neurogenic commitment, particularly under redox imbalance. Pharmacologically, this distinction is critical: cortisol is inactivated by 11β-HSD2, whereas synthetic glucocorticoids are not, potentially prolonging GR activation and thereby favoring progenitor maintenance over differentiation.

Building on this, Nürnberg et al. [65] investigated whether endogenous GC signaling contributes to NPC maintenance and differentiation. In hiPSC-derived NPCs, GR activation—whether by exogenous DEX or endogenous ligands—promoted proliferation and survival. This effect was abolished by the GR antagonist mifepristone, which not only suppressed proliferation but also increased the proportion of differentiated neurons. These findings suggest that sustained GR activation maintains progenitor identity, whereas its downregulation may be required to allow neurogenesis to proceed. Expression of CYP11B1 in these cultures supports the possibility of local cortisol synthesis, adding complexity to the balance between endogenous signaling and pharmacological exposure.

Beyond intrinsic signaling, GC also modulate NPC fate through their interaction with immune pathways. Preynat-Seauve et al. [63] showed that DEX reduced IFN-γ secretion by NK cells co-cultured with hESC-derived NPCs, although it did not prevent NPC death caused by NK activity. More strikingly, DEX impaired the neuronal maturation of NPCs, as shown by reduced neurite outgrowth, decreased total cell body area, and increased prevalence of intermediate progenitor phenotypes. These effects occurred independently of immune cells and were similarly observed with cyclosporin A, suggesting that immunosuppressive agents can directly interfere with NPC differentiation trajectories.

Although our transcriptomic analysis was not limited to NPCs, the resulting transcriptional patterns echo core features reported in NPC-focused studies. We observed upregulation of genes such as *ID3*, *MSX1*, and *ZBTB16*, associated with biological processes including negative regulation of cell differentiation, regulation of cell population proliferation, and response to hormone stimulus. In parallel, there was consistent downregulation of genes like *NFIA*, *NFIB, UCHL1*, and *NEUROD6*, linked to axon development, forebrain development, and neuronal maturation. This molecular profile suggests a convergence toward impaired differentiation, sustained progenitor-like states, and altered responsiveness to environmental cues. These findings align with the experimental evidence from Ninomiya et al. [64], Nürnberg et al. [65], and Preynat-Seauve et al. [63], who reported increased NPC proliferation, accumulation of intermediate progenitor phenotypes, and disrupted neuronal maturation following GR activation. Together, these observations indicate that early glucocorticoid exposure may impose a durable transcriptional imprint across developing neural systems, potentially contributing to altered neurodevelopmental trajectories relevant to psychiatric vulnerability.

#### Neurons and Astrocytes

GR signaling remains active throughout neuronal differentiation and continues to regulate key transcriptional programs in mature cells. In line with this, Babaniyi et al. [68] extended the evidence for GR-mediated control into postmitotic neurons, showing that DEX induces FKBP5 and PER1 expression in a dose-dependent manner, with saturation at 100 nM. Co-treatment with the GR antagonist mifepristone inhibited *FKBP5* induction, confirming the specificity of the GR pathway. These findings parallel earlier observations in NPCs by Nürnberg et al. [65], suggesting that GR signaling remains transcriptionally active throughout neural maturation. In our analysis, *FKBP5* and *PER1* were upregulated, associated respectively with nuclear receptor signaling, interferon responses, and MECP2-regulated neuronal networks, as well as circadian rhythm and MAPK-related pathways. This transcriptional profile supports the notion that GC exposure reprograms stress, immune, and rhythmic signaling pathways in human neurons.

Yet, this GR-dependent transcriptional activity is not uniformly detected across neuronal models. Lieberman et al. [66] reported a modest induction of *FKBP5* and unchanged *NR3C1* expression in iPSC-derived forebrain-lineage neurons. Seah et al. [34] similarly observed no *FKBP5* upregulation following DEX or cortisol treatment in NGN2-induced glutamatergic neurons from donors with PTSD and controls, despite robust PTSD-specific transcriptional responses to cortisol at lower doses. Interestingly, the transcriptomic reanalysis revealed downregulation of *NR3C1*, with enrichment in GO terms strongly associated with neuroendocrine and stress-related processes, including ‘response to glucocorticoid’, ‘glucocorticoid receptor pathway’, ‘regulation of hormone levels’, ‘behavior’, and ‘regulation of neuron apoptotic process’. While Lieberman et al. [66] observed *NR3C1* downregulation only in fibroblasts, our results raise the possibility that certain human neuronal populations also suppress GR expression transcriptionally in response to GC exposure. This aligns with prior evidence linking reduced *NR3C1* expression to impaired HPA axis feedback and increased vulnerability to mood disorders, including MDD [74, 75].

Astrocytes play an increasingly recognized role in mediating the effects of GCs on the developing brain. Their transcriptional responses reflect not only GR sensitivity but also disease-specific vulnerability and stage-specific plasticity. Heard et al. [67] analyzed astrocytes derived from individuals with MDD and reported consistent upregulation of *FKBP5* following both acute and chronic cortisol exposure—mirroring patterns previously observed in neurons [68] and NPCs [65]. However, MDD astrocytes exhibited a distinct transcriptional profile compared to controls, with altered expression of genes related to GPCR signaling, ion transport, and synaptic modulation. Notably, *MAOA* was upregulated and *NTRK2* downregulated—two genes previously implicated in MDD pathophysiology [76, 77]. In this reanalysis, *MAOA* expression varied across datasets, whereas *NTRK2* showed consistent upregulation, particularly in studies involving prolonged GC exposure. This discrepancy may reflect differences in cellular composition, donor background, exposure duration, or compensatory mechanisms.

Together, these findings underscore that GC exposure induces cell type-specific transcriptional programs in neurons and astrocytes, which may converge to alter synaptic function, neuroendocrine regulation, and stress reactivity—key pathophysiological mechanisms associated with psychiatric vulnerability.

#### 3D models

Three-dimensional human neural models offer a valuable platform to study how GCs impact neurodevelopmental trajectories in the context of psychiatric disorders. These models enable the investigation of GR-mediated transcriptional and signaling changes that may underlie long-term vulnerability to stress-related conditions. Recent studies employing neural organoids have identified recurrent patterns of transcriptional remodeling induced by GC exposure. Dony et al. [72] demonstrated that GCs bias organoid differentiation toward an inhibitory neuronal fate, driven by the upregulation of the transcription factor *PBX3*. Concurrently, *YBX1*, *NFIA*, *NFIB*, and *EGR1*—factors associated with excitatory neuron development and progenitor maintenance—were consistently downregulated. These effects were observed across different iPSC lines and cell types, suggesting a robust and conserved GR-dependent reprogramming. Both Dony and Krontira et al. [33] reported *PBX3* upregulation and *YBX1* downregulation in response to chronic GC exposure, highlighting their role in stress-induced transcriptional shifts.

Krontira et al. [33] further dissected GC-regulated transcriptional programs and identified four factors consistently induced by DEX: *TSC22D3*, *KLF9*, *ZBTB16*, and *HEYL*. Among them, *ZBTB16* modulated key regulators of proliferation and differentiation, such as *TP53* and *CRABP1*, particularly in PAX6-positive progenitors, suggesting a role in fate specification under GC exposure. Although originally validated in fetal mouse cortex, the transcriptional signature associated with *ZBTB16* has also been detected in hiPSCs-derived neural models [33, 68, 72], reinforcing its potential relevance across models.

While most studies have focused on transcriptional regulators, GCs also induce changes at the metabolic and structural level in 3D models. For instance, Notaras et al. [70] identified N-acetylglutamine as a specific metabolic marker of chronic cortisol treatment and reported increased expression of proteins such as NDUFB9, PBDC1, RBM28, ROCK2, and SLC16A2, along with decreased levels of ARMC9, INTS9, VIRMA, and NUP210. Many of these are involved in mitochondrial function, RNA processing, and nuclear transport, suggesting that chronic cortisol signaling disrupts fundamental cellular processes beyond transcriptional control. These findings provide further support for the hypothesis that GCs affect multiple layers of neurodevelopmental regulation, potentially contributing to structural and functional alterations observed in stress-related psychiatric disorders.

Mechanistic insights into GC-mediated signaling disruption have also revealed a consistent repression of the Wnt pathway, a key regulator of progenitor proliferation and neurogenesis. Moors et al. [69] identified DEX-induced suppression of Wnt signaling in human NPCs, driven by GR-mediated upregulation of DKK1, a canonical Wnt antagonist. This led to decreased β-catenin and downregulation of targets such as Cyclin D1 and ID2, impairing stem cell maintenance. In line with these findings, the reanalysis revealed widespread Wnt-related alterations following GC exposure. Upregulated terms included negative regulators such as ZNRF3, LGR4, TLE4, and *SOX4*, while downregulated genes included canonical effectors like *CCND2*, *TCF7L2*, and *WNT2B*. Notably, genes such as *TLE1*, *CCND1*, and *WNT2B* appeared across both up- and downregulated pathways, suggesting context-dependent and cell type–specific modulation of Wnt activity [33, 68, 72].

In addition to its role in progenitor regulation, dysregulation of Wnt signaling has also been implicated in the pathophysiology of psychiatric disorders. In MDD, GSK3β, a downstream effector of the Wnt pathway, shows increased expression and altered activity in the hippocampus and prefrontal cortex of affected individuals [78, 79], and polymorphisms in GSK3β have been associated with hippocampal structural changes [80]. In schizophrenia, neurons derived from hiPSCs of patients exhibit reduced neurite complexity and synaptic markers, alongside altered expression of multiple Wnt-related genes, including *WNT2B*, *WNT7A*, *TCF4*, and *LEF1* [81]. Notably, in GC-treated organoid datasets, *TCF4* and *LEF1* were consistently downregulated across independent studies [33, 48, 72], while *WNT2B* exhibited context-dependent modulation—being strongly upregulated in some conditions [72]. These findings highlight transcriptional convergence between GC-induced Wnt dysregulation and pathways implicated in psychiatric disorders, reinforcing the relevance of 3D neural models for investigating stress-related neurodevelopmental vulnerability.

#### Exposure Duration on Glucocorticoid Responses

Transcriptomic comparisons in human neural models have shown that the duration of GC exposure can markedly influence the molecular response. For example, Heard et al. [67] compared acute (24 h) and chronic (7 days) cortisol treatment in iPSC-derived astrocytes and found that chronic exposure dysregulated nearly twice as many genes as acute exposure and induced a large set of transcripts uniquely affected under prolonged treatment. Gene Ontology (GO) analysis revealed that these chronic-specific genes were enriched for pathways related to cell adhesion, extracellular matrix organization, tyrosine kinase signaling, gliogenesis, and regulation of cell death—processes not significantly altered after acute exposure. Such findings suggest that, even within the same cell type and stimulus, exposure duration can engage distinct transcriptional programs.

**Fig. 3:**
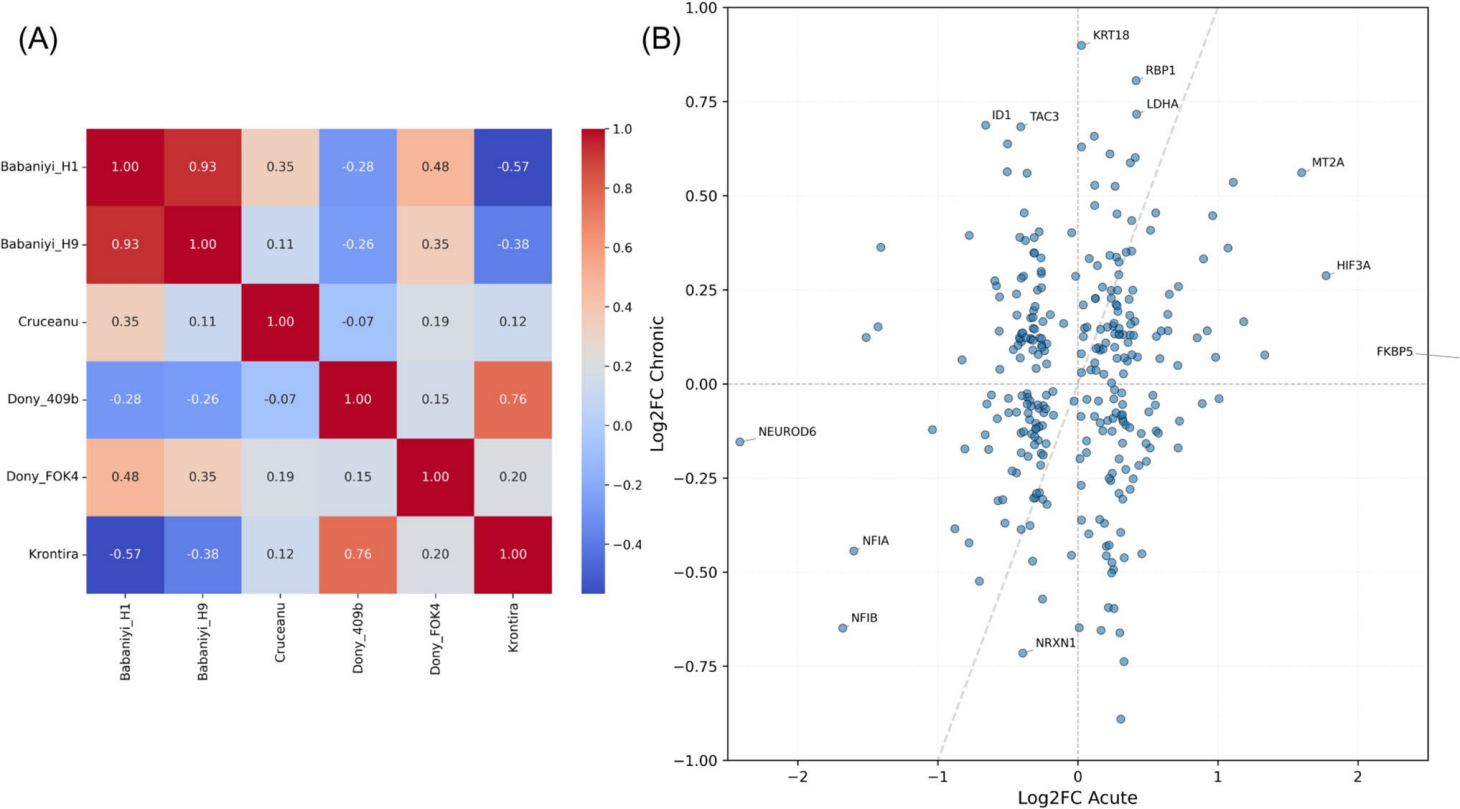
Comparative analysis of acute vs. chronic DEX exposure in human neural models. **A** Pearson correlation heatmap across six datasets derived from three acute (Babaniyi_H1, Babaniyi_H9, Cruceanu) and three chronic (Dony_409b, Dony_FOK4, Krontira) DEX exposure conditions. **B** Scatter plot comparing gene-level log₂FC values from acute versus chronic datasets, highlighting the most transcriptionally divergent genes across exposure paradigms

To investigate whether a similar divergence occurs in human neural in vitro models treated with DEX, we reanalyzed six RNA-seq datasets derived from four independent studies [33, 48, 68, 72], encompassing different neural cell types, exposure durations, and experimental designs (Fig. 3). While Babaniyi et al. [68] datasets comprised mixed neural cultures containing both neurons and astrocytes, the other studies employed 3D organoid models, from which we selectively analyzed neuronal populations based on the available gene expression matrices. This decision aimed to minimize variability arising from heterogeneous cell-type composition across datasets and to focus on the lineage most consistently represented among all models. Neurons constitute the primary cellular interface for GC signaling in the central nervous system, directly mediating transcriptional, synaptic, and stress-adaptive responses to DEX. By restricting our reanalysis to neuronal signatures, we sought to capture core and comparable GC-responsive mechanisms across studies that differ in experimental architecture and cellular diversity, while still retaining Babaniyi et al. [68] datasets to anchor the analysis in physiologically mixed neural contexts.

The acute group included: (i) Babaniyi et al. [68] treated hESC-derived neural cultures (H1 and H9 lines) with 1 μM DEX for 6 h; (ii) Cruceanu et al. [48] exposed hiPSC-derived hCOs from three independent lines to 100 nM DEX for 12 h. The chronic group included: (i) Dony et al. [72], who treated unguided hCOs derived from two hiPSC lines with 100 nM DEX for 10 consecutive days starting at day 60 of differentiation; and (ii) Krontira et al. [33], who similarly applied 100 nM DEX for 10 days starting at day 60 to hiPSC-derived hCOs. It is important to note that these datasets differ not only in exposure duration but also in DEX concentration—particularly in the acute group (1 μM vs. 100 nM). Such differences can alter receptor saturation levels, downstream signaling kinetics, and the breadth of transcriptional responses, making it difficult to disentangle whether observed divergences are driven by exposure time, concentration, or both. This represents a key limitation when interpreting time-dependent effects across studies. Furthermore, it is important to emphasize that our reanalysis utilized pre-normalized expression matrices as provided by the original authors. While these datasets were subjected to study-specific normalization procedures across different sequencing platforms, we addressed potential batch and platform effects by applying uniform statistical rigor (FDR < 0.05 and |Log2FC|> 0.1) and focusing exclusively on a consensus signature of genes present in at least two independent datasets.

Correlation analysis across datasets (Fig. 3A) revealed overall low to moderate concordance among studies, although strong correlations were observed between specific datasets sharing identical exposure paradigms, such as Babaniyi_H1 and Babaniyi_H9 (*r* = 0.93) or independent chronic models like Dony and Krontira (*r* = 0.76). In this context, the correlation between aggregated acute and chronic DEX conditions was low but statistically significant (*r* = 0.124, *p* = 3.45 × 10⁻^2^). To move beyond the limitations of gene-level variability and technical noise, we performed a pathway-level comparison to identify robust biological programs. While a core set of genes remained responsive across both paradigms, the chronic group engaged a much broader transcriptional landscape, suggesting that sustained GC exposure recruits additional regulatory networks that are not yet active or significant under acute conditions.

GO enrichment analysis revealed a clear temporal shift in biological priorities. The acute-responsive genes were heavily dominated by canonical glucocorticoid signaling, metabolic regulation, and rapid stress-adaptation pathways—reflecting the immediate genomic response to GR activation. Conversely, the chronic-associated genes were enriched for pathways related to long-term structural remodeling, including gliogenesis, cell–matrix adhesion, and extracellular matrix organization (Supplementary Fig. 2). This divergence suggests that while the acute response is geared toward immediate homeostasis, chronic DEX exposure triggers a transition toward developmental and architectural modifications in neural tissues. Such processes, particularly the dysregulation of glial-related pathways and structural plasticity, are frequently implicated in the pathophysiology of chronic stress-related psychiatric disorders [82, 83], where sustained cortisol elevation is thought to drive maladaptive neural changes.

Although this pattern is consistent with the notion that acute and chronic DEX exposure may engage partially distinct transcriptional responses, the variability observed across datasets—even within the same exposure category—precludes a definitive attribution of these differences to exposure duration alone. Instead, the results point to substantial cross-study heterogeneity, likely driven by a combination of biological and technical factors, including differences in culture systems, cell-type composition, DEX concentration, exposure timing, and analytical pipelines. Accordingly, the correlation analysis is best interpreted as providing suggestive evidence of temporal specificity in GC-responsive transcriptional programs, highlighting the need for improved cross-study normalization or experimental designs that directly contrast acute and chronic conditions within matched systems.

At the gene level, the acute–chronic comparison (Fig. 3B) further illustrates this tendency toward temporal differentiation, with a consensus of 291 genes displaying distinct regulatory behaviors across conditions. Acute exposure was associated with preferential upregulation of canonical GC-responsive genes such as *FKBP5*, *MT2A*, and *HIF3A*, consistent with rapid GR-mediated transcriptional activation and cellular stress responses. In contrast, chronic exposure showed relative enrichment of genes including *KRT18*, *RBP1*, and *TAC3*, which have been implicated in cytoskeletal organization and signaling pathways. A small subset of genes—*NFIA*, *NFIB*, and *NEUROD6*—was consistently downregulated across the most significant records of both paradigms, suggesting the presence of shared repressive responses that are less sensitive to exposure duration. Detailed gene-level expression values, corresponding studies, and FDR-adjusted significance metrics are provided in Supplementary Table 3, and a comprehensive breakdown of the GO enrichment results for both signatures is provided in Supplementary Table 4.

Taken together, these analyses suggest that acute and chronic DEX exposure may preferentially engage different transcriptional features, as reflected by gene-level trends in the acute–chronic scatter plot, while also revealing substantial overlap and variability across studies. Rather than defining discrete transcriptional programs, these results are consistent with a model in which GC signaling unfolds along a temporal continuum, with early and sustained exposures tending to bias gene expression toward partially distinct, yet highly context-dependent, regulatory states in human neural in vitro systems.

#### Functional Convergence with Psychiatric Disorders and Systems-Level Regulatory Framework

**Fig. 4:**
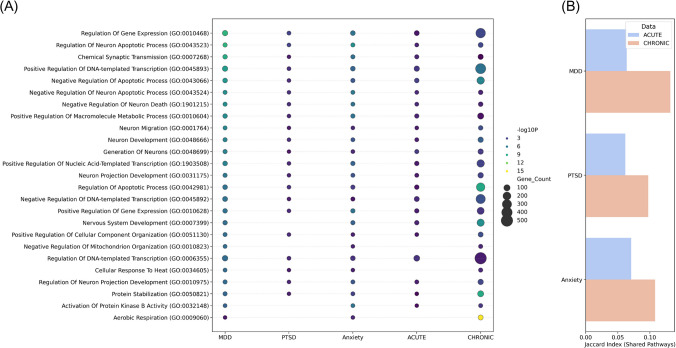
Comparative enrichment and translational overlap of acute vs. chronic DEX exposure in human neural models. **A** Dot plot representing the enrichment of Gene Ontology terms across psychiatric clinical categories and experimental paradigms. Clinical signatures for MDD, PTSD, and Anxiety were curated and filtered from the DisGeNET database to ensure the inclusion of robustly associated gene-disease associations. The dot size represents the gene count, while the color gradient indicates statistical significance. **B** Bar chart illustrating the Jaccard Index for shared functional pathways. Across all psychiatric conditions, the chronic DEX exposure (orange) exhibits a consistently higher Jaccard Index compared to acute treatment (blue), demonstrating a superior molecular convergence with the filtered DisGeNET clinical landscapes

To investigate the potential clinical relevance of the identified consensus signatures, we performed a comparative functional convergence analysis integrating DEX-responsive genes with curated disease-associated gene sets from the DisGeNET database (v7.0), accessed via the Enrichr platform. Specifically, we extracted consensus gene lists from our acute and chronic reanalysis groups—following the previously described selection criteria (FDR < 0.05 and |Log2FC|> 0.1) and independent study consensus—and compared them against high-confidence gene signatures associated with MDD, PTSD, and anxiety disorders, given their strong mechanistic links to stress-responsive transcriptional programs and impaired glucocorticoid sensitivity. We employed a Jaccard Index-based approach to quantify the similarity between the biological pathways enriched in our filtered datasets and those implicated in psychiatric pathophysiology (Fig. 4). The Jaccard Index was calculated as the ratio of the intersection to the union of enriched GO terms between each DEX exposure group and the respective psychiatric disorder.

The analysis revealed that while both exposure paradigms overlap with stress-related disorders, chronic DEX exposure consistently displayed a higher Jaccard Index across all clinical categories, peaking at ~ 0.12 for MDD (Fig. 4, B). This indicates that the chronic signature more closely mirrors the pathophysiology of long-term affective disorders. This convergence is particularly driven by a large cluster of pathways related to the control of cellular homeostasis and transcription. Specifically, the chronic signature exhibited substantially higher gene counts in categories such as Regulation of Gene Expression, Regulation of Apoptotic Process, and both Positive and Negative Regulation of DNA-templated Transcription.

Furthermore, the dot plot (Fig. 4, A) highlights that both acute and chronic paradigms significantly converge with all three psychiatric disorders across a wide range of neurobiological pathways. Beyond the stable core of responsiveness in Neuron Development and Chemical Synaptic Transmission, the chronic group showed a dominant signature in both Positive and Negative Regulation of Apoptotic Process. The increased gene count in these regulatory and cell-death pathways during chronic exposure suggests that sustained GC stimulation triggers a complex, systems-level reprogramming. This process likely involves a fine-tuned balance between transcriptional activation and inhibition, alongside the modulation of cell survival programs, which may be central to the progression of stress-induced psychiatric conditions.

To identify the regulatory drivers of these signatures, we performed a systems-level enrichment analysis of transcription factor binding evidence using ChEA 2022 and ENCODE/ChEA Consensus libraries. The results show a clear divergence in the transcriptional machinery engaged by different exposure durations.

Acute exposure was significantly enriched for targets of the Polycomb Repressive Complex 2 (PRC2), including *SUZ12*, *EZH2*, *RNF2*, and *RING1B* (Supplementary Fig. 3 A). We also found enrichment for core regulators such as *SOX2* and *NANOG*. This suggests that the primary acute response to DEX is characterized by active chromatin remodeling and epigenetic silencing of developmental programs.

In contrast, the chronic consensus signature showed a shift toward the basal transcriptional machinery and cell cycle control, with significant enrichment for *TAF1*, *MYC*, and *E2F1* targets. The high statistical significance of *TAF1* and consistent enrichment across MYC ChIP-seq datasets (Supplementary Fig. 3B) indicate that sustained GC exposure triggers a systemic reprogramming of RNA metabolism and cellular biogenesis.

Notably, several TFs showed evidence of regulation in both conditions, but with substantially higher significance in the chronic group. This reflects a temporal expansion of the GC-responsive network: the initial response, driven by direct GR targets, evolves into a systems-level cascade recruiting a wider network of co-regulators. These changes likely sustain long-term architectural and functional alterations in neurons. While these convergences suggest how DEX-induced changes relate to disease mechanisms, these results represent statistical tendencies in cellular models and do not constitute direct proof of clinical causality. Further details on these regulatory enrichments are provided in Supplementary Table 5.

## Discussion

GCs modulate key neurodevelopmental processes in hiPSC-derived neural models, including proliferation, neuronal differentiation, synaptic maturation, and glial function [64, 65]. When such changes occur during early developmental stages—as those captured in studies using NPCs or immature neuronal cultures—they can disrupt the formation and refinement of neural circuits, which are critical for cognitive and emotional regulation. In NPC-focused models, GC exposure has been shown to alter cell cycle progression, impair neuronal differentiation, and dysregulate transcriptional programs involved in axon guidance and synapse formation [64, 65]. Despite the recognized vulnerability of NPCs to GC exposure, evidence from hiPSC-derived NPCs generated from individuals with stress-related psychiatric conditions remains limited, representing a critical gap in linking patient-specific neurodevelopmental trajectories to disease-relevant molecular mechanisms.

Although many of these transcriptional alterations were described in NPCs, similar GC-driven changes have been observed in more differentiated cell types and complex systems, including astrocytes [67, 68], neurons [34, 71], and 3D models [33, 72]. Across these systems, GCs consistently modulated stress-responsive genes such as *FKBP5*, *TSC22D3*, and *ZBTB16*, alongside pathways related to immune signaling and structural remodeling. However, cell-type-specific differences emerged in both the direction and magnitude of these changes: astrocytic models tended to show stronger induction of gliogenesis and inflammatory mediators [68], whereas neuronal cultures exhibited more pronounced synaptic gene downregulation [34, 71]. Organoids, in turn, captured both glial and neuronal signatures, with chronic exposure revealing sustained effects on developmental and stress-related networks [33, 72]. This convergence across models reinforces the idea that key GC-responsive genes are shared across cell types, while their downstream functional consequences may be highly context-dependent.

Our comparative reanalysis further demonstrates that these transcriptional responses are not merely scaled versions of the same signal but represent a fundamental shift in regulatory engagement over time. This temporal divergence—characterized by a transition from acute PRC2-mediated epigenetic remodeling (*SUZ12*, *EZH2*) to a systemic co-opting of the basal transcriptional machinery (*TAF1*, *MYC*) under chronic conditions—suggests that sustained GC exposure induces a profound, maladaptive reprogramming of cellular biogenesis. This parallels in vivo evidence showing that short-term GR activation triggers adaptive signaling, whereas prolonged stimulation induces GC resistance and maladaptive remodeling [84, 85]. Available evidence indicates that exposure duration critically shapes both transcriptional and functional outcomes: acute stimulation elicits transient modulation of synaptic plasticity and cytoskeletal remodeling, whereas prolonged exposure leads to sustained transcriptional reprogramming, reduced BDNF–TrkB signaling, and altered progenitor differentiation [23, 86, 87].

The translational relevance of this shift is underscored by its functional convergence with psychiatric pathophysiology. While both paradigms overlap with stress-related pathways, the chronic signature mirrors the molecular landscape of MDD and Anxiety with significantly higher precision, as evidenced by a higher Jaccard Index across all clinical categories. Specifically, the convergence in pathways regulating gene expression and apoptotic processes indicates that chronic GC exposure captures the persistent molecular alterations defining clinical vulnerability more accurately than acute pulses. By bridging cellular-level signaling with systems-level genomics, these findings provide a robust framework for using human neural models to investigate disease-relevant mechanisms.

The translational relevance of these transcriptional signatures is further supported by their convergence with known genetic risk loci for psychiatric disorders. Evidence indicates that polymorphisms within key HPA axis regulators, such as *NR3C1*, *FKBP5*, and *CRHR1*, contribute significantly to individual susceptibility to MDD, PTSD, and suicidal behavior [88, 89]. For instance, specific SNPs in the *NR3C1* gene (e.g., rs258747 and rs41423247) have been shown to interact with environmental stressors, such as traumatic life events and low social support, to increase PTSD risk by 3.26-fold [89, 90]. Similarly, risk haplotypes in *FKBP5* (including rs3800373 and rs1360780) are associated with altered HPA axis sensitivity and higher risk for suicide attempts, particularly when interacting with childhood abuse [88, 91]. Notably, the influence of these genetic variants on clinical outcomes is often observed through gene-environment interactions, where the association with disorder severity is significantly amplified in the presence of traumatic life events or chronic stress [90, 91]. By demonstrating that GCs drive profound changes in the expression of these same regulatory genes across different neural systems, this reanalysis provides a functional context for how glucocorticoid-driven signaling may interface with established GWAS risk loci. This alignment reinforces the utility of integrating hiPSC-derived transcriptomic data with genetic findings to better capture the dynamic mechanisms through which environmental stressors and genetic predisposition converge in psychiatric pathophysiology [88, 89].

Differences in GC type, concentration, and exposure period further modulate these effects. The pharmacokinetic properties of cortisol and DEX—rapidly metabolized versus more stable and longer-acting, respectively—affect the duration and amplitude of GR activation and consequently the persistence of downstream transcriptional responses. Together with variations in GR/MR expression, receptor sensitivity, and cellular composition, these factors likely account for much of the cross-study heterogeneity in GC responsiveness. Such mechanistic and methodological variability has broader conceptual implications for the field, underscoring how experimental heterogeneity can fundamentally alter biological interpretation. To improve reproducibility, harmonized exposure frameworks are required, including standardized definitions for “acute” and “chronic” paradigms and consistent reporting of concentration, duration, and receptor context. Implementing such consistency would allow both transient and sustained GC-responsive programs to be captured within unified experimental systems, enabling clearer comparisons, and strengthening translational relevance.

Beyond these methodological factors, developmental stage itself represents an additional layer of variability influencing GC sensitivity, as receptor availability and downstream signaling capacity evolve during neural maturation. Evidence from postmortem and in vitro developmental studies illustrates this principle. Sinclair et al. [92] and Lieberman et al. [66] demonstrated that FKBP5 and NR3C1 expression increases across human brain development, with significantly higher levels in adult frontal cortex compared to immature neural tissue. This developmental upregulation has been interpreted as a functional maturation of the GC response system, potentially rendering cortical neurons more sensitive to stress hormones in adulthood. Lieberman et al. [66] further showed that 6–7-week-old iPSC-derived neural cultures exhibit FKBP5 and NR3C1 expression profiles most similar to first-trimester human forebrain [81] indicating that such cultures may be too immature to fully replicate genotype-dependent FKBP5 induction following GC exposure. This developmental context is critical when interpreting GC effects in vitro, as exposure during stages of low FKBP5/NR3C1 expression may engage only a subset of stress-response pathways active in the mature brain, with implications for modeling stress-related psychiatric conditions, including PTSD, anxiety, and MDD.

In addition to developmental stage and methodological variability, the biological sex of the donor cell lines represents a critical factor in GC responsiveness. Our review of the literature reveals a significant divergence in how sex is reported and balanced across studies. While some investigations utilized exclusively male cohorts (e.g., Nürnberg et al. [65]; Seah et al. [34]) or female cohorts (e.g., Heard et al. [67]), others employed mixed-sex lines with varying ratios, often showing a higher proportion of male (Notaras et al. [70]) or female (Lieberman et al. [66]) donors. Furthermore, several studies included in this review did not explicitly report the sex of the utilized lines, and others integrated both male and female rodent littermates for primary culture validations (Byun et al. [71]). In our reanalysis, this imbalance is also evident: the acute group utilized a combination of male and female-derived lines (Cruceanu et al. [48]), whereas the chronic consensus signature was derived from predominantly female lines, such as 409b2 and FOK4 [72]. Given the established sexual dimorphism in HPA axis regulation and stress-related transcriptional programs in the human brain, the lack of standardized sex-specific reporting and the prevalence of single-sex paradigms limit the generalizability of the findings. These gaps prevent a clear determination of whether the observed temporal divergence between acute and chronic exposure is modulated by sex-dependent factors. Future research must prioritize sex-balanced experimental designs and transparent metadata reporting to ensure that in vitro models accurately reflect the interindividual variability observed in psychiatric disorders.

Collectively, these findings position hiPSC-derived neural systems as a relevant experimental framework for investigating GC effects across human neural cell types and developmental stages, while emphasizing the importance of integrating postmortem molecular data from stress-related psychiatric conditions to strengthen translational validity. The convergence between GC-responsive pathways in vitro and molecular alterations reported in PTSD, anxiety, and MDD—including GR signaling, immune regulation, and synaptic remodeling—supports the use of these models for identifying disease-relevant molecular mechanisms. Advancing this field will require studies incorporating multiple hiPSC-derived cell types from individuals with stress-related psychiatric disorders, using standardized protocols for GC type, concentration, and exposure duration, as well as consistent molecular readouts, to reduce variability and enable more direct comparisons between in vitro findings and human brain pathology.

## Concluding Remarks

The reviewed studies collectively delineate how GCs influence human neural development, revealing consistent regulation of pathways related to neurogenesis, immune signaling, and structural organization of neural circuits. Our integrative reanalysis further demonstrates that these molecular changes significantly converge with the genomic landscapes of MDD and PTSD, particularly under chronic exposure. Across NPCs, neurons, and glial cells in both 2D and 3D systems, GCs modulate processes such as forebrain and axon development, cell-cycle control, cytokine signaling, and projection morphogenesis, while also affecting networks linked to neurodegeneration and stress hormone signaling. Despite these advances, a major conceptual gap remains: most investigations have examined either acute or chronic paradigms in isolation, limiting the integration of temporal dimensions into the understanding of GC effects. By mapping the regulatory drivers of these signatures, we identified a temporal shift from acute PRC2-mediated epigenetic silencing to a broad, systemic reprogramming of the transcriptional machinery in chronic states. Recognizing this dual pattern—partially divergent responses built upon a common molecular foundation—advances current models of GC signaling and offers new insight into how exposure duration can shape adaptive versus persistent cellular outcomes.

Building on these insights, future research should prioritize mechanistic integration across time, cell type, and regulatory layer. While our findings emphasize human-specific iPSC models to ensure translational relevance to psychiatric vulnerability, the identification of highly conserved regulatory hubs, such as GR-binding patterns and chromatin remodelers, provides a bridge to cross-species mechanistic validation. Temporal multi-omics approaches—combining transcriptomic, epigenomic, and chromatin-accessibility profiling could unravel how early transcriptional activation, chromatin remodeling, and receptor occupancy interact to determine response persistence. Incorporating sex-dependent and receptor-specific analyses will further clarify how GR and MR signaling dynamics contribute to interindividual variability in stress susceptibility. Furthermore, bridging these human cellular trajectories with in vivo functional readouts will enable a more comprehensive mapping of GC signaling—from immediate, reversible activation to long-term maladaptive remodeling—within the complex architecture of the brain.

Advancing this agenda will require harmonized experimental standards for GC type, concentration, and duration, together with transparent reporting of receptor context and developmental stage. These steps will enhance reproducibility and comparability across studies while providing a deeper, temporally resolved understanding of how glucocorticoid signaling sculpts neural development, plasticity, and vulnerability to psychiatric disorders.

## Supplementary Information

Below is the link to the electronic supplementary material.

ESM 1 Supplementary Material 1 (PDF 1.13 MB)

ESM 2 Supplementary Material 2 (XLSX 287 KB)

ESM 3 Supplementary Material 3 (XLSX 42.0 KB)

ESM 4 Supplementary Material 4 (XLSX 82.0 KB)

ESM 5 Supplementary Material 5 (XLSX 345 KB)

ESM 6 Supplementary Material 6 (XLSX 325 KB)

## Data Availability

The datasets reanalyzed in this study were obtained from publicly available repositories cited in the reviewed articles. All processed data and original Python scripts used for comparative transcriptomic integration, including functional enrichment and correlation analyses, are openly available in the GitHub repository: (https://github.com/eloizadalmolin/DEX_neural_models).
