# Supplementary material for: Glucocorticoid Signaling in PSC-Derived Neural Systems to Elucidate Mechanisms of Stress-Induced Psychiatric Vulnerability
Source: Mol Neurobiol. 2026 Jul 7;63(1):748. doi: 10.1007/s12035-026-06041-1 (PMC13341972; doi:10.1007/s12035-026-06041-1)
Supplement: Supplementary file 1 — Supplementary Material 1 (PDF 1.13 MB) [file 12035_2026_6041_MOESM1_ESM.pdf]

Molecular Neurobiology

Glucocorticoid Signaling in PSC-Derived Neural Systems to Elucidate Mechanisms of Stress-Induced Psychiatric Vulnerability

Eloiza Adriane Dal Molin, Manuella Pinto Kaster, Juliana Minardi Nascimento*

Department of Biochemistry, School of Biological Sciences, Federal University of Santa Catarina, Florianopolis, SC, Brazil

*Corresponding Author:

Juliana Minardi Nascimento, Department of Biochemistry, Federal University of Santa Catarina, Florianopolis, SC, Brazil; email: juminardi@gmail.com

Supplementary Information content:

Supplementary Figure 1
Supplementary Figure 2
Supplementary Figure 3
Supplementary Table 1 (.xlsx file)
Supplementary Table 2 (.xlsx file)
Supplementary Table 3 (.xlsx file)
Supplementary Table 4 (.xlsx file)
Supplementary Table 5 (.xlsx file)

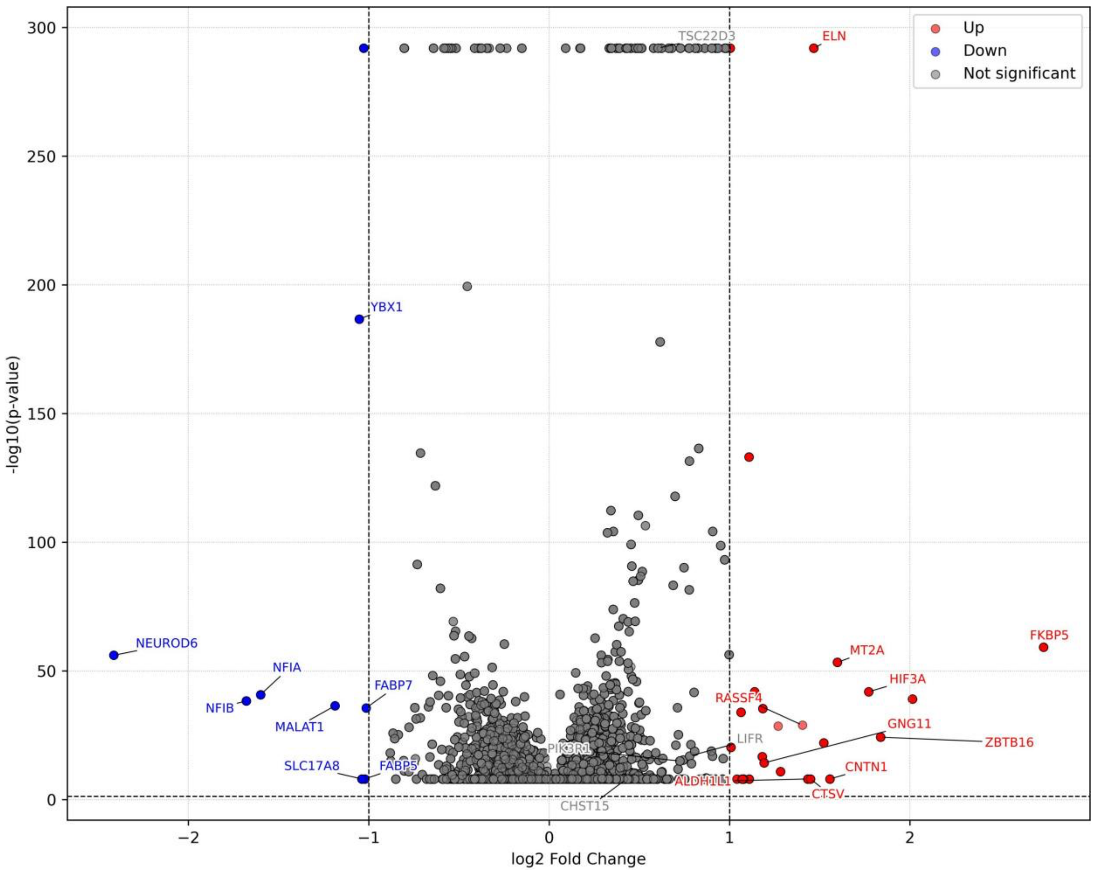

Supplementary Figure 1. Volcano plot integrating effect size and cross-study consistency of differential gene expression. Volcano plot depicting $\log_2$ fold change versus statistical significance (–$\log_{10}$ p-value) for genes identified across the integrated transcriptomic datasets of dexamethasone exposure. Vertical dashed lines indicate the fold-change threshold ($|\log_2 FC| = 1$), and the horizontal dashed line denotes the nominal significance cutoff. Upregulated genes are shown in red and downregulated genes in blue, while genes not meeting significance criteria are shown in grey. The plot highlights both the most strongly up- and downregulated genes and transcripts that show consistent regulation in the same direction across all six datasets, irrespective of individual effect size. Consistently upregulated genes—*FKBP5, HIF3A, RASSF4, TSC22D3, PIK3R1,* and *CHST15*—are explicitly labeled, reflecting reproducible glucocorticoid-responsive regulation across studies.

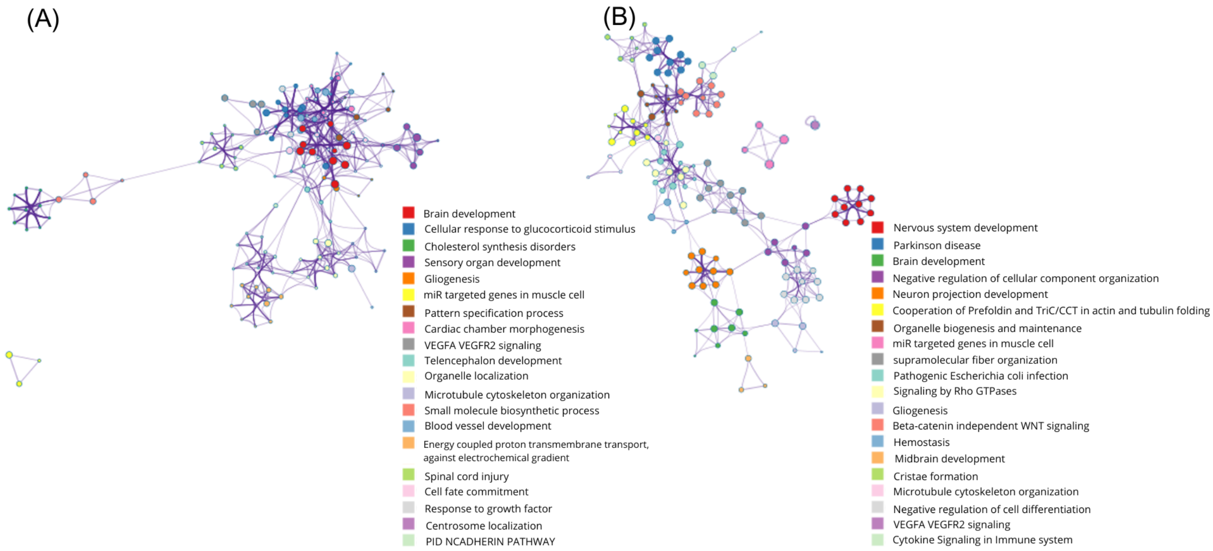

Supplementary Figure 2. Supplementary Figure 2. Temporal shift in biological pathways following acute and chronic DEX exposure. Enrichment networks of differentially expressed genes (DEGs) were generated using Metascape, illustrating the biological priorities under (A) acute and (B) chronic regimens. The acute response is dominated by canonical glucocorticoid signaling and rapid metabolic adaptation, whereas the chronic signature reveals a transition toward long-term structural remodeling, extracellular matrix organization, and gliogenesis. Each node represents an enriched term, colored by its cluster ID; nodes with the same color are closely related, with edges connecting terms that share a significant number of genes.

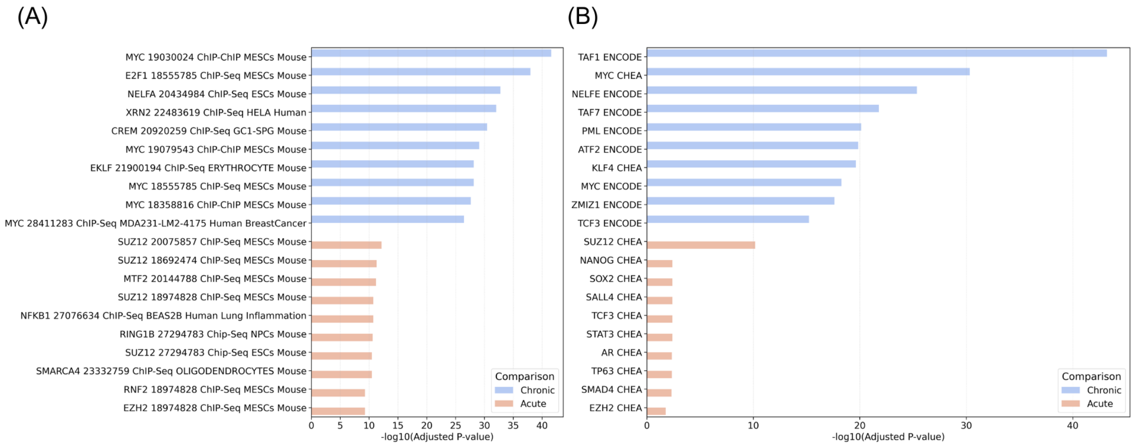

Supplementary Figure 3. Regulatory driver analysis of acute and chronic DEX signatures. Enrichment analysis of transcription factor (TF) targets based on (A) individual ChIP-seq experiments and (B) ENCODE/ChEA Consensus libraries. Bars represent the statistical significance for chronic (blue) and acute (orange) consensus signatures. Acute exposure is primarily characterized by the recruitment of Polycomb Repressive Complex 2 (PRC2) components (e.g., *SUZ12, EZH2*) and pluripotency factors (*SOX2, NANOG*), indicating active epigenetic remodeling. Conversely, the chronic signature shows a robust shift toward the basal transcriptional machinery (*TAF1*) and cell cycle/biogenesis regulators (*MYC, E2F1*). Detailed enrichment scores are provided in Supplementary Table 5.

Table Legends

Supplementary Table 1: Gene set enrichment analysis results summarizing significantly enriched biological processes and pathways modulated by dexamethasone (DEX) exposure. The table includes ontology terms, associated gene counts, enrichment statistics, and significance metrics derived from Metascape analyses [1].

Supplementary Table 2: Functional annotation of differentially expressed genes (DEGs) following DEX exposure across datasets. For each gene, the table reports associated biological processes and pathways, expression values, adjusted p-values, and the contributing studies, as identified through enrichment and differential expression analyses.

Supplementary Table 3: Gene-level expression data for the acute versus chronic DEX exposure comparison. The table includes fold-change values, statistical significance, corresponding cell types, and study sources, enabling evaluation of both consistency and context-dependent variability across datasets.

Supplementary Table 4: A detailed breakdown of the functional enrichment analysis for both acute and chronic signatures. The table lists enriched Biological Processes, Molecular Functions, and Cellular Components, including terms related to canonical glucocorticoid signaling and metabolic regulation (predominant in the acute response) and structural remodeling, extracellular matrix organization, and gliogenesis (predominant in the chronic response). Metrics provided include gene counts, fold enrichment, and adjusted p-values.

Supplementary Table 5: Detailed enrichment scores for transcription factor (TF) binding evidence derived from ChEA 2022 and ENCODE/ChEA Consensus libraries. This table specifies the regulatory targets for the acute response (enriched for PRC2 components such as *SUZ12* and *EZH2*) and the chronic signature (enriched for basal transcriptional machinery, including *TAF1* and *MYC*). It includes the complete list of regulatory targets, statistical significance for each TF, and evidence across different ChIP-seq datasets.
